# First Draft Genome Sequence of a Human *Coxiella burnetii* Isolate, Originating from the Largest Q Fever Outbreak Ever Reported, the Netherlands, 2007 to 2010

**DOI:** 10.1128/genomeA.00445-15

**Published:** 2015-05-07

**Authors:** J. A. Hammerl, K. Mertens, L. D. Sprague, V. H. Hackert, J. Buijs, C. J. Hoebe, K. Henning, H. Neubauer, S. Al Dahouk

**Affiliations:** a Department of Biological Safety, Federal Institute for Risk Assessment, Berlin, Germany; b Friedrich-Loeffler-Institut, Federal Research Institute for Animal Health, Institute for Bacterial Infections and Zoonoses, National Reference Laboratory for Fever, Jena, Germany; c Public Health Service South Limburg, Department of Sexual Health, Infectious Diseases, and Environmental Health, Sittard-Geleen, the Netherlands; d Department of Internal Medicine, Atrium Medical Center, Heerlen, the Netherlands; e School of Public Health and Primary Care (CAPHRI), Maastricht University Medical Centre (MUMC+), Maastricht, the Netherlands; f Department of Internal Medicine III, RWTH Aachen University, Aachen, Germany

## Abstract

In 2009, *Coxiella burnetii* caused a large regional outbreak of Q fever in South Limburg, the Netherlands. Here, we announce the genome draft sequence of a human *C. burnetii* isolate, strain NL-Limburg, originating from this outbreak, including a brief summary of the genome’s general features.

## GENOME ANNOUNCEMENT

Q fever is a zoonotic disease caused by *Coxiella burnetii*. Acute human infections are often asymptomatic or characterized by mild flu-like symptoms. Though rarely observed, chronic Q fever, mostly affecting patients with heart valve or vascular anomalies, may be fatal (1). Published genome sequences revealed a close relationship among *C. burnetii* isolates originating from various sources (2–6). This sequencing project was performed with the *C. burnetii* strain NL-Limburg, isolated by cell culture from a surgical specimen of an abdominal aortic aneurysm in 2011. The patient had shown late sequelae from an asymptomatic primary infection and ultimately died from multiple organ failure. This fatality could be epidemiologically linked to a single-point source Q fever outbreak on a dairy goat farm located next to Voerendaal, South Limburg, the Netherlands, in 2009 (7). This outbreak totaled 253 laboratory-confirmed human cases in the region, while the actual number of residents who incurred infection was estimated to be 9,000. Genome determination of NL-Limburg may contribute to a better understanding of the biology, evolution, and virulence of this pathotype, which showed very high attack rates both in humans and animals and was associated with the largest Q fever outbreak ever reported, comprising 4,026 human cases in the Netherlands (8).

Axenic cultivation of NL-Limburg was performed in modified acidified citrate cysteine medium (ACCM-2) for 7 days at 37°C (2.5% O_2_, 5% CO_2_) (9). Isolation of genomic DNA was conducted by incubating bacteria with proteinase K/SDS at 56°C for 18 h, followed by phenol-chloroform treatment and ethanol precipitation (10). Genome sequencing on the PacBio_RS system was performed by GATC (Konstanz, Germany). A *de novo* genome assembly based on 92,760 reads (*N*_50_ length, 7.022; mean length and score, 4.691 and 0.86, respectively) from one SMRT cell was developed by using the SMRT Analysis (version 2.3.0) software (Pacific Biosciences, USA), resulting in a sequence coverage of 60- to 160-fold per consensus base. The draft genome sequence contains 2,214,254 bp, allocated to five contigs (C00, 1,373,721 bp; C01, 292,414 bp; CO2, 25,635 bp; C03, 472,758 bp; C04, 49,726 bp) with an average G+C content of 42.8%. Genome comparisons using different BLAST algorithms from NCBI (11) indicate that the closest relatives of the NL-Limburg isolate are RSA_331, RSA_493, and Z3055; differences are mainly based on single nucleotide polymorphisms. Contig04 comprises the complete sequence of a 37.4-kb pQpH1-like plasmid-encoding type IV secretion effector (12). Initial genome annotation was performed with the automated NCBI Prokaryotic Genome Annotation Pipeline (http://www.ncbi.nlm.nih.gov/genome/annotation_prok) and revealed 2,401 genes, 2,097 CDS, 257 pseudogenes, 43 tRNAs, three rRNAs, and one noncoding RNA. Comparable to other *C. burnetii* genomes, the majority of the gene products are involved in metabolism of amino acids and carbohydrates, ribosomal structure and biogenesis, cell wall and membrane biogenesis, replication, recombination, and repair. Genome finishing is in progress and will contribute to the final assessment of DNA regions linked to pathogenicity of this highly virulent strain.

### Nucleotide sequence accession number. 

The draft genome sequence of NL-Limburg has been deposited in GenBank under the accession number JZWL00000000.
